# Temporal Dynamics of Airborne Concentrations of *Ganoderma* Basidiospores and Their Relationship with Environmental Conditions in Oil Palm (*Elaeis guineensis*)

**DOI:** 10.3390/jof10070479

**Published:** 2024-07-12

**Authors:** Juan Manuel López-Vásquez, Sandra Yulieth Castillo, León Franky Zúñiga, Greicy Andrea Sarria, Anuar Morales-Rodríguez

**Affiliations:** Pest and Disease Program, Colombian Oil Palm Research Center—Cenipalma, Bogotá 111121, Colombia; sycastillo@cenipalma.org (S.Y.C.); lzuniga@cenipalma.org (L.F.Z.); gsarria@cenipalma.org (G.A.S.); amorales@cenipalma.org (A.M.-R.)

**Keywords:** oil palm, Basal Stem Rot, *Ganoderma*, aerobiology

## Abstract

Basal Stem Rot (BSR), caused by *Ganoderma* spp., is one of the most important emerging diseases of oil palm in Colombia and is so far restricted to only two producing areas in the country. However, despite the controls established to prevent its spread to new areas, containment has not been possible. This study aimed to understand BSR’s propagation mechanisms and related environmental conditions by measuring *Ganoderma* basidiospores’ concentrations at various heights using four 7-day Burkard volumetric samplers in a heavily affected plantation. Meteorological data, including solar radiation, temperature, humidity, precipitation, and wind speed, were also recorded. Analysis revealed higher basidiospore concentrations below 4 m, peaking at 02:00 h, with increased levels towards the study’s end. Spore concentrations were not directly influenced by temperature, humidity, or precipitation, but showed higher releases during drier periods. A significant correlation was found between wind speed and spore concentration, particularly below 1.5 m/s, though higher speeds might aid long-distance pathogen spread. This study highlights the complexity of BSR propagation and the need for continued monitoring and research to manage its impact on Colombia’s oil palm industry.

## 1. Introduction

The production of vegetable oils continues to be one of the fastest-growing agro-industrial chains worldwide due to high demand in international markets. Globally, approximately 217.68 million metric tons of vegetable oils are produced annually, with palm oil contributing 35.8% of the total (78.06 million metric tons), making it one of the most significant sectors in the global production of these oils [1]. Colombia is characterized as a competitive country in the production of consumer goods at both regional and international levels. Currently, it is the leading producer of palm oil in the Americas and ranks fourth globally, with a planted area of 576,799 hectares, a production of 1,768,013 tons, and a national average yield of 3.63 tons per hectare [2]. As a result, oil palm cultivation (*Elaeis guineensis* Jacq.) in Colombia has been consolidating as one of the leading sectors in the national agro-industry due to its significant growth potential, impact on the agricultural economy, and contribution to the country’s social development [3].

However, in recent years, the sustainability of oil palm cultivation in the country has been threatened by a series of lethal or difficult-to-manage diseases that hinder the rapid growth of the sector. One of the limiting diseases of oil palm and most widely distributed worldwide is Basal Stem Rot (BSR) [4]. BSR is considered one of the main threats to oil palm production worldwide as it can cause the death of infected palms and significantly reduce both yield and oil quality [5]. In countries like Malaysia and Indonesia, major producers of crude palm oil, this disease can reduce yields by between 50% and 80%, particularly in replanted plantations on fields previously affected by the disease [6]. Published research on the identification of the causal agent of BSR has demonstrated that depending on the area of influence, there is a greater affinity towards a particular species of *Ganoderma*. Thus, in southeast Asia, the presence of at least six *Ganoderma* species has been reported, including *G. miniatocinctum*, *G. chalceum*, *G. tornatum*, *G. xylonoides*, *G. zonatum*, and *G. boninense*, with the latter being the most predominant species [7,8,9]; in Colombia, the only species identified so far from the isolates obtained has been *G. zonatum* [10]. However, in Colombia, the possibility that other *Ganoderma* species are associated with the development of the disease cannot be ruled out, as is the case in southeast Asia [11].

In Colombia, similar to what occurs in Malaysia and Indonesia, there are two types of symptomatology caused by species of the genus *Ganoderma*: Basal Stem Rot (BSR) characterized by the formation of basidiocarps in the lower third of the stem, and Upper Stem Rot (USR) characterized by the formation of basidiocarps in the upper third of the stem. Despite both symptomatologies sharing the decomposition of internal tissues, field observations on dissected palms with USR suggest that the development of this symptomatology is not related to the infective process of BSR in oil palm [12]. In Colombia, USR is a less frequent manifestation compared to BSR; however, in Indonesia, severe cases have been recorded in which a complete fracture of the stem occurs [13,14,15].

For a long time, it has been assumed that *Ganoderma* dissemination in established plantations mainly occurs through root contact between diseased and healthy palms, and that the mere presence of this pathogen in the soil becomes a serious issue, especially in second- and third-generation renewal lots, possibly due to root transmission from remnants of infected tissue from previous plantations [16]. However, genetic diversity studies of *G. boninense* in Malaysia and Papua New Guinea revealed considerable diversity in oil palm plantations according to genetic studies conducted using mitochondrial DNA markers, mating alleles, and somatic compatibility [15,17,18]. Similar results have been reported in Colombia with isolates of *Ganoderma*, whose somatic compatibility assessments indicated that all evaluated isolates were genetically different despite many of them being isolated from the same lot [19].

These results imply that infections by separate genotypes likely arose through unique sexual recombination exclusively via interaction with the pathogen’s basidiospores. This argument was corroborated by Rees et al. [18] who demonstrated that direct infection by basidiospores through cuts in the stem and indirect infection through roots are more common than previously believed, compared to infection derived from vegetative clonal propagation. More recently, Pilotti et al. [20], based on an inoculum source study, proposed modifying the disease cycle originally conceived by Flood et al. [21]. In this modification, basidiospores play a more significant role in the infectious cycle of the disease by being considered a direct source of inoculum in the field, in addition to infected stem tissue and roots present in the soil.

Despite these advances, many uncertainties persist regarding the behavior of *Ganoderma* basidiospores in the air, their concentration depending on altitudinal gradient, their seasonal fluctuation at different time scales, and the effect of meteorological conditions on these concentrations [11,22,23,24]. Therefore, the main objective of this study was to evaluate the concentrations of airborne *Ganoderma* basidiospores at different altitudinal gradients and their relationship with the timing of capture and meteorological conditions to clarify the processes involved in pathogen dispersion in oil palm and facilitate the development of more effective and efficient management and control strategies.

## 2. Materials and Methods

### 2.1. Description of the Study Site

**Figure 1:**
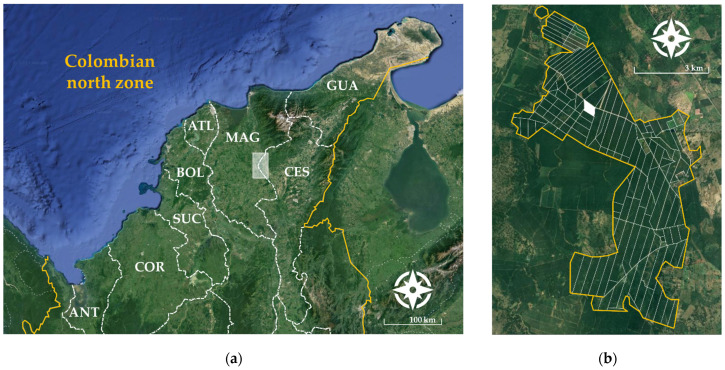
Spatial location of the study site. (**a**) Departments comprising the northern Colombian zone. ANT: Antioquia; COR: Córdoba; SUC: Sucre; BOL: Bolívar; ATL: Atlántico; MAG: Magdalena; CES: Cesar; GUA: Guajira. White box shows the geographical location at GREMCA S.A. plantation. (**b**) Distribution of all oil palm lots at GREMCA S.A. plantation. White box shows the spatial location of the selected lot according to the highest accumulated incidence of BSR recorded at the time of the study.

This research was carried out at GREMCA S.A. plantation located in the municipality of El Copey (Cesar) in Colombian north zone, where BSR has been recognized as a relevant issue (Figure 1a). This area is characterized by belonging to a region of tropical dry forest, with a bimodal seasonality of rains in the months of April/May and September/November. The study site was selected based on a temporal epidemiological analysis of historical BSR records, focused on identifying the lot with the highest accumulated incidence during the last production cycle [25]. The selected plot has an area of 14.23 hectares and has established oil palms of African origin (*E. guineensis*), cultivar Deli × La Mé. The age of the palms at the time of evaluation was 17 years, with an average height of 12 m above ground level (Figure 1b).

### 2.2. Monitoring of Basidiospore Concentrations

**Figure 2:**
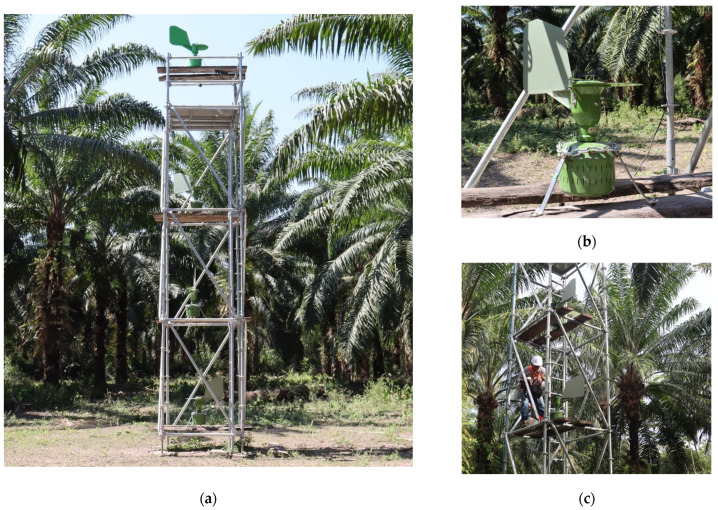
Field arrangement of volumetric samplers. (**a**) Scaffold-type platform divided into 4 vertical sections. (**b**) Detail of the Burkard^®^ volumetric sampler used in the study. (**c**) Maintenance, calibration, and replacement procedure of Melinex tapes every 7 days.

Within the selected plot and at a free point after removing a palm due to BSR, a scaffold-like platform that was divided into 4 vertical sections arranged at heights of 1, 4, 7, and 10 m was installed (Figure 2a). In each vertical section, a 7-day volumetric spore sampler from Burkard^®^ (Rickmansworth, Hertfordshire, UK) was placed. The samplers operated from 1 June 2022, to 31 May 2023, at a constant flow rate of 10 L of air per minute (Figure 2b). Each volumetric sampler used a Melinex tape coated with Vaseline for spore capture. After 7 days of sampling, the tapes were removed from the equipment and placed in sealed containers for transport to the Phytopathology Laboratory of Cenipalma (Figure 2c). There, in the laboratory, and with the help of a metric ruler, 48 mm long segments were cut, each representing one day of sampling. Finally, each tape segment was affixed to a glass slide with glycerin jelly (50 mL of glycerin, 7 g of gelatin, 1 g of phenol, and 42 mL of distilled water).

For spore quantification, before fixing the Melinex tape, the glass slides were marked with 24 vertical lines at 2 mm intervals, corresponding to each hour of the evaluated day. The tape was examined by assessing the center of each vertical transect in the direction of the drum’s movement inside the volumetric sampler. Identification and quantification of *Ganoderma* spores were conducted using a 40× light optical microscope with the assistance of a spore and pollen atlas in the air [26]. For this procedure, it was not necessary to use spore staining techniques because *Ganoderma* basidiospores are pigmented, which facilitated their identification. The *Ganoderma* basidiospores were identified as ellipsoid-shaped spores, slightly equinulate, golden yellow to light brown, with dimensions ranging between 10.6 (9.6–11.3) μm and 6.0 (5.5–6.6) μm. The counts were corrected to compensate for the sampled area and recorded as the number of basidiospores per cubic meter (spores/m^3^) of sampled air following the Hirst methodology [27].

### 2.3. Meteorological Data and Statistical Analysis

All meteorological records were obtained using the statistical package R version 2023.09.1 (R Core Team 2021) nasapower [28]. This package allows for the free download of a large amount of meteorological data from the NASAPOWER program (National Aeronautics and Space Administration Prediction of Worldwide Energy Resource, Washington, DC, USA). Meteorological data querying in the nasapower package was conducted from the same geographic point where the volumetric samplers were installed in the field (Latitude: 10.1269; Longitude: −74.0464; Altitude: 196 m above sea level) and during the same monitoring dates between 00:00 h on 1 June 2022 and 24:00 h on 31 May 2023. All consulted meteorological parameters corresponded to hourly records of the following variables: solar radiation (Mj/m^2^), temperature (°C), relative humidity (%), precipitation (mm), wind speed at 2 and 10 m above ground level (m/s), and wind direction at 10 m above ground level (°). A statistical correlation analysis was performed considering the spore concentration variable (spores/m^3^) as dependent and the meteorological variables as independent. The non-parametric Spearman correlation test (Rho) was applied for each of the 12 months evaluated, as it was verified that the obtained spore concentrations do not follow normal distribution models. Subsequently, the significance level was calculated for confidence intervals of 95% (*) and 99% (**). Monthly records of wind speed and direction were analyzed and visualized using WRPLOT View software version 8.0.2 (Lakes Environmental Software).

### 2.4. Field Disease Monitoring

From the beginning to the end of the trial, a sanitary census (palm by palm) was conducted in all lots of the plantation with a frequency not exceeding 30 days. This task was performed by a team that was trained to visually identify *Ganoderma* basidiocarps, located in both the upper third of the affected palms (USR) and the lower third of the base (BSR). The characteristics of the identified basidiocarps include a sessile basidiome ranging in shape from reniform to irregular; a light brown pileus surface that is lacquered, opaque to semi-opaque, and slightly rough; and a rounded, glabrous margin that is pure white during growth. An electronic template was used to record the monitoring data, registering healthy palms (0) or diseased ones (1), providing the exact date of detection and the spatial location of the palm detected through pre-established geographical coordinates. The analysis of disease monitoring records did not consider the severity level of USR or BSR due to the inability to quantify the internal tissue affected by *Ganoderma* and the high subjectivity in assessing external secondary symptoms. Spatial information analysis was performed by generating monthly distribution maps of detected cases of the disease categorized into five rings with radii of 2000, 4000, 6000, 8000, and 10,000 m from the sampling point.

## 3. Results

### 3.1. Capture Height

**Figure 3:**
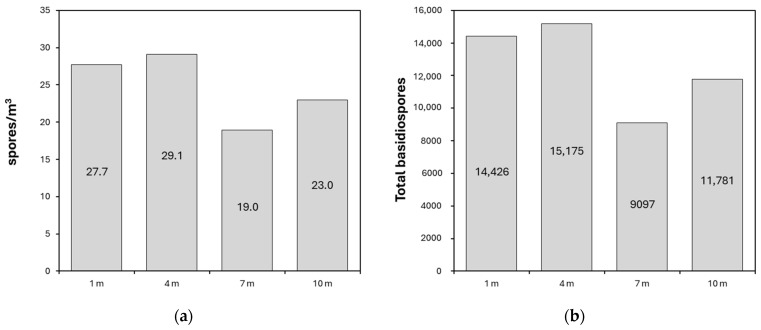
Concentrations and total *Ganoderma* basidiospores captured at 1, 4, 7, and 10 m above ground level. (**a**) Average concentration of basidiospores (spores/m^3^) captured. (**b**) Total basidiospores quantified over 365 days of evaluation.

During the observation period, a total of 50,479 *Ganoderma* basidiospores were captured across the different altitude gradients evaluated. The highest concentration of basidiospores was obtained at a height of 4 m, followed in order by 1, 10, and 7 m (Figure 3a,b). The low capture of basidiospores observed at a height of 7 m may be due to a technical failure in the timing mechanism of the volumetric sampler during the period of highest basidiospore concentration in the air. This issue caused the sampler to be inactive for 8.6% of the total sampling time (762 h out of 8760 h, considering 24 h per day over 365 days) due to corrective maintenance. Despite this, statistical analyses conducted on the estimated concentrations did not show statistical significance.

### 3.2. Capture Periodicity

**Figure 4:**
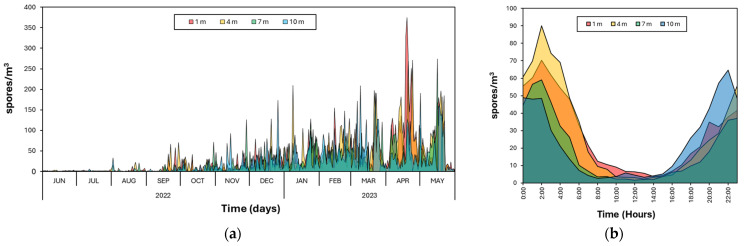
Patterns of periodicity of *Ganoderma* basidiospores according to their temporality. (**a**) Periodicity of basidiospore concentration captured per day at four different heights. (**b**) Periodicity of basidiospore concentration captured per hour at four different heights. The values given in graph (**b**) are basidiospore concentrations captured at the same time of day between 1 June 2022 and 31 May 2023.

The assessment of the periodicity corresponding to the concentration of basidiospores in the air according to the day of capture fluctuated throughout the observation period. This fluctuation ranged from 0 spores/m^3^ on 6 June 2022 to a peak of 1.8 × 10^2^ spores/m^3^ on 19 April 2023. Initially, the fluctuation was low in the first month evaluated, June 2022, with an average of 0.5 spores/m^3^. However, the concentration in the following months gradually increased as the evaluation time progressed, reaching a maximum peak of 71.3 spores/m^3^ in April 2023. Subsequently, this concentration decreased to 57.6 spores/m^3^ in May 2023. Regarding the concentrations recorded at different heights, the highest concentration of basidiospores observed in a day occurred at one meter height with 3.7 × 10^2^ spores/m^3^ on 19 April 2023, followed by 7, 4, and 10 m heights, with 2.7 × 10^2^ spores/m^3^ on 16 May 2023, 2.7 × 10^2^ spores/m^3^ on 24 April 2023, and 2.0 × 10^2^ spores/m^3^ on 9 March 2023, respectively (Figure 4a).

The assessment of the periodicity corresponding to the concentration of basidiospores according to the hours of the day indicated a pattern of higher capture during nighttime hours, between 18:00 and 5:00 h, with a total of 42,876 basidiospores captured, corresponding to 84.93% of the total captured. Daytime hours, between 06:00 and 17:00 h, showed lower captures with a total of 7603 basidiospores captured, corresponding to 15.05% of the total captured. The hour with the highest concentration recorded in the air was at 02:00 h, with an average of 64.7 spores/m^3^, while the hour with the lowest concentration recorded in the air was at 13:00 h, with an average of 2.8 spores/m^3^. Regarding the concentrations recorded at different heights, the highest concentration of basidiospores observed in an hour occurred at 4 m height with an average of 90.0 spores/m^3^ at 02:00 h, followed by 1, 10, and 7 m with 70.4 spores/m^3^ at 02:00 h, 64.4 spores/m^3^ at 22:00 h, and 59.1 spores/m^3^ at 02:00 h, respectively (Figure 4b).

### 3.3. Climatic Correlation

According to Spearman correlation coefficients, meteorological parameters such as solar radiation, temperature, relative humidity, and precipitation did not show statistically significant relationships with basidiospore concentrations in the air in any of the 12 months evaluated. Despite the relatively weak correlations between monthly basidiospore concentration values and the mentioned parameters, a pattern in the type of relationship between the variables evaluated was observed. This relationship was positive for the solar radiation and temperature variables in 8 and 9 months, respectively, out of the 12 months evaluated. In contrast, relative humidity and precipitation showed a negative relationship in 9 and 8 months, respectively, out of the 12 months evaluated (Table 1).

**Figure 5:**
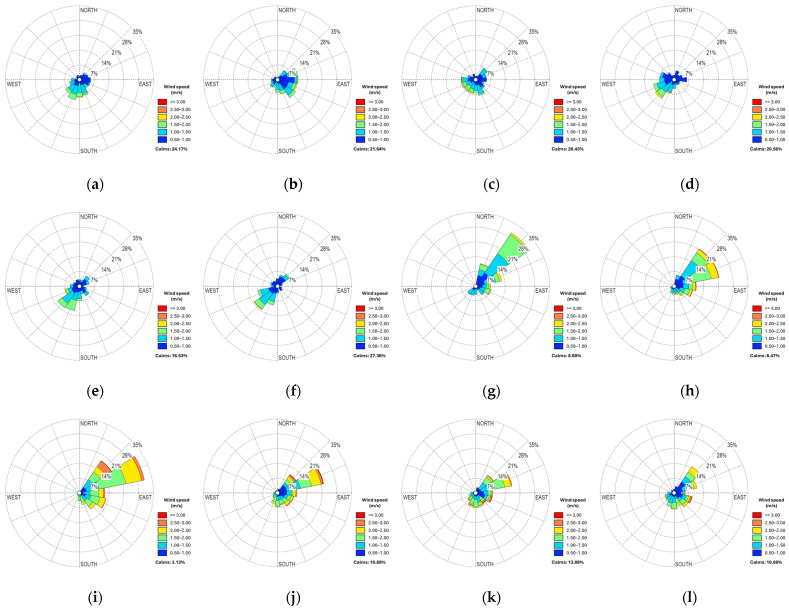
Wind speed and direction measured at 10 m above ground level. (**a**–**g**) correspond to the months of June, July, August, September, October, November, and December 2022, respectively; (**h**–**l**) correspond to the months of January, February, March, April, and May 2023, respectively. All wind direction and speed graphs were configured from the origin of winds to the same magnitude scale.

On the other hand, wind speed measured at 2 and 10 m above ground level was predominant when airborne basidiospore concentrations were grouped seasonally. As a result, the coefficients obtained regarding wind speed measured at 2 and 10 m showed a positive and significant correlation between the months of December 2022 and January 2023. According to the results from Spearman’s statistical correlation tests, wind speed at 10 m above ground level was the only meteorological parameter that explained the behavior of airborne basidiospore concentrations with greater significance, especially in December and January. Additionally, during these same months, there was an abrupt change in wind direction, transitioning from a predominance of southwest winds at the sampling point between June and December 2022 to a completely opposite northeast wind predominance at the sampling point between January and May 2023 (Figure 5).

### 3.4. Field Disease Monitoring with Respect to the Sampling Point

**Figure 6:**
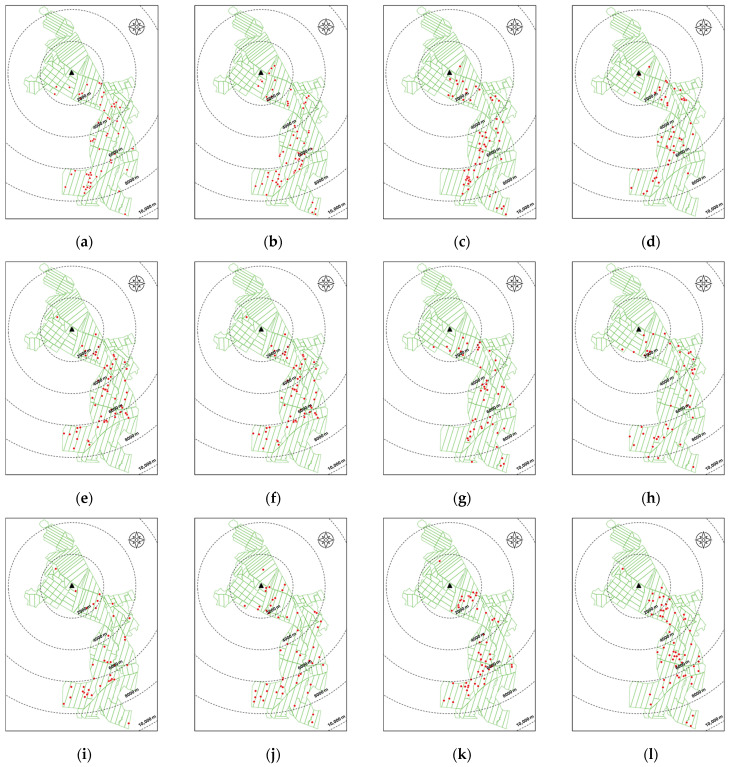
Spatial distribution of palms detected with characteristic symptoms of Basal Stem Rot. (**a**–**g**) correspond to the months of June, July, August, September, October, November, and December 2022, respectively; (**h**–**l**) correspond to the months of January, February, March, April, and May 2023, respectively. The black triangle on the graphs corresponds to the sampling point, and the red dots correspond to the location of the palms detected with BSR.

During the 12 months of evaluation, 680 cases of disease were recorded, with an average of 56.6 cases per month, peaking at 73 cases detected in July 2022 and reaching a minimum of 39 in February 2023, without a clear pattern of peak months. Regarding the temporal and spatial distribution of detected cases, the highest number of cases (215) was observed within a distance no greater than 8000 m from the sampling point, followed in order by distances of 6000, 2000, 4000, and 10,000, with 189, 126, 124, and 26 cases detected, respectively (Figure 6).

## 4. Discussion

The highest concentration of basidiospores was found at heights between 1 and 4 m, while at heights between 7 and 10 m, the concentration was lower. This difference suggests a pattern of higher spore concentration at lower heights, possibly related to the height of the fruiting bodies on affected palms. Similar results were reported by Jedryczka et al. [29] who found concentrations of *Ganoderma* basidiospores 1.6 to 6 times higher at a height of 1 m than those captured at 18 m. In our study, although *Ganoderma* concentrations varied at different altitudinal gradients, we did not find statistically significant differences. Furthermore, it was verified that at 4, 7, and 10 m in height, it is possible to find, on some occasions, basidiospore concentrations equal to or greater than those found at ground level. This result is relevant as evidence of the possible origin of USR, as until now, studies revolved around the discontinuity, both physical and genetic, between USR and BSR infections. In particular, dissections performed on affected palms showed no relationship [12,13,14,15], and high genetic diversity of *Ganoderma* isolates was found within plantations [15,17,18], even in isolates obtained from the same lot [19]. In light of these results, it is evident that basidiospores play a key role in the development of both manifestations of stem infection. In the case of BSR, the soil becomes an ideal reservoir of basidiospores for direct root colonization or through colonized residues [20]. In the case of USR, it is possible that it arises from a significant amount of airborne basidiospores deposited in the leaf axils or directly in wounds caused by leaf cutting [18]. However, the success of any *Ganoderma* infection will depend solely and exclusively on the occurrence and mating of haploid basidiospores to form infectious heterokaryons, and this clearly has a greater probability of occurring in the soil than in the stems of the palms, hence the low incidence of USR compared to BSR.

In this study, basidiospore concentrations fluctuated over the 12-month evaluation period, showing an increasing trend towards the end of the period. The peak of highest daily basidiospore concentration was recorded in the last months of evaluation, with 3.7 × 10^2^ spores/m^3^ captured at one meter height. Research conducted by Rees et al. [18] on *G. boninense* revealed peaks of up to 1.1 × 10^4^ spores/m^3^ using water agar plates placed at a height of two meters. Although the monitoring and quantification method used by Rees et al. [18] differed from the one used in this study, it is possible to record large quantities of basidiospores considering that the release of up to 2.0 × 10^6^ spores/min has been estimated in individual basidiocarps of *G. boninense* with a surface area of 5 cm^2^ [30]. This also partly explains the difference in aggressiveness between *Ganoderma* isolates from Malaysia and Colombia. According to monitoring reports, in Colombia BSR does not exceed 5% of cumulative incidence in second-generation lots [25], unlike reports from affected plantations in Malaysia, where the disease can even reach 30% of cumulative incidence in lots of the same generation [31].

Regarding the periodicity of basidiospore concentration according to the hours of the day, the results showed a nocturnal pattern of higher release, with 84.93% of the total spores captured between 18:00 and 05:00 h, peaking at 02:00 h. This same circadian rhythm of basidiospore release by *Ganoderma* fungi has been reported by other authors, who found high concentrations of basidiospores during the night from 22:00 to 06:00 h, with a peak capture around midnight [32,33]. However, Rees et al. [18] recently found that although the highest concentration of *G. boninense* basidiospores occurs during the night, the peak concentration actually occurs at 19:00 h. In light of the evident formation of circadian discharge patterns by *Ganoderma* fungi, Haard and Kramer [34] suggested that environmental factors such as temperature, precipitation, relative humidity, and soil moisture availability could influence the circadian pattern of spore discharge. However, Ho and Nawawi [32] demonstrated that even when *G. boninense* basidiocarps are subjected to a constant temperature of 26 to 28 °C and a relative humidity between 85 and 90%, the circadian rhythm of spore release between the nocturnal maximum and diurnal minimum remained unchanged. The explanation for these phenomena could be related to the adaptive and evolutionary processes inherent to the fungus. One piece of evidence is that the highest rate of spore release for *Ganoderma* occurs during nighttime hours rather than during the day, as direct UV radiation exposure could decrease the fungus’s survival chances, spore viability, and the pathogen’s infection capacity.

Regardless of the circadian rhythm, the relationship of meteorological parameters, such as temperature, relative humidity, and precipitation, that are possibly involved in a higher rate of basidiospore release into the air did not show significant values in the *Spearman* correlation coefficients. However, a pattern could be identified in the type of relationship between the evaluated variables, where solar radiation and temperature showed positive correlation coefficients, unlike relative humidity and precipitation, which showed negative correlation coefficients in most of the months evaluated. This demonstrates that ideal environmental conditions for increased release may be more related to dry periods than wet periods. This assertion is reinforced by the fact that the highest daily concentration of basidiospores in the air (1.8 × 10^2^ spores/m^3^) was recorded precisely during a dry period, between 18:00 h on 19 April and 05:00 h on 20 April 2023, with an average nighttime temperature of 26.8 °C and a relative humidity of 70.7%, with no precipitation recorded in the last 48 h. This trend of the fungus towards drier environments has been previously documented by Ingold [35], who noted that the *Ganoderma* genus appears to form tolerant basidiocarps (distinctive among basidiomycetes) that can not only survive under very dry conditions but also continue to release their basidiospores despite drought. This affinity has been such that airborne activities of *Ganoderma* basidiospores have even been recorded in desert environments [36], and a significant number of publications show positive correlation coefficients with a high level of significance between temperature increase and *Ganoderma* concentrations in the air [29,37,38]. However, despite reports of a higher concentration of *Ganoderma* basidiospores in the air in the absence of precipitation and highly significant negative correlation coefficients [29,37,39], the same does not apply to the relative humidity parameter. Still now, there is no defined consensus on the effects (positive or negative) of this parameter on *Ganoderma* basidiospore concentrations in the air. An example of this is a number of publications that, although showing a very low level of significance, demonstrate a positive relationship between relative humidity and *Ganoderma* basidiospore concentrations in the air, [29,40,41,42], while there are other publications showing the opposite [37,38,43].

On the other hand, wind speed between December 2022 and January 2023 was the only meteorological parameter that showed significant correlation coefficients with *Ganoderma* basidiospore concentrations in the air, and this correlation was even stronger when they were grouped seasonally. This positive and significant relationship was observed when the concentration averaged over 20 spores/m^3^ in the air. According to wind zoning studies conducted in the northern region of Colombia, the strong wind season lasts for four months, typically covering the months of December, January, February, and March, with a peak in February [44]. Our results showed a positive and significant correlation at the beginning of the wind season, in December and January. However, this trend did not persist in the following months of higher speed; in fact, the relationship was negative in February and weak in March. At the start of the wind season, the average speed was 1.1 m/s (max. 1.7 m/s) in December and 1.2 m/s (max. 2.0 m/s) in January. In contrast, in February and March, the wind speed increased to 1.5 m/s (max. 2.2 m/s) and 1.2 m/s (2.0 m/s), respectively. These results suggest that although wind speeds of 1.5 m/s may contribute to basidiospore release, higher readings in this speed range could have the opposite effect on local basidiospore concentrations. However, they could significantly influence the pathogen’s dissemination over longer distances. This aligns with findings reported by Grinn-Gofrón et al. [37], who noted that locally, the maximum spore concentrations corresponded to very low wind speed conditions. However, they observed extremely high concentrations of *Ganoderma* basidiospores in the air at sites where sources were very distant (>900 km), which could have resulted from the transport of additional spores from remote sources via higher wind speeds.

Throughout the evaluation period, the plantation recorded a significant number of palms with active *Ganoderma* basidiocarps (average of 56.6 cases/month), with some of them detected even at distances less than 100 m from the sampling point, indicating a constant source of inoculum in the sampling area. However, it is noteworthy that despite the spore traps being installed in a disease hotspot plot, the area with the highest number of cases was in the ring 6000 to 8000 m from the capture point. This could possibly be due to the disease spreading towards younger lots located to the south of the plantation. It is important to highlight that many of the detections carried out by trained plantation personnel are made in the early stages of basidiocarp development (primordia or button stage), which may not align with patterns of higher concentrations of basidiospores in the air. This also explains the observation that although the wind direction in the first six months of evaluation originated from areas with positive detections of palms affected by *Ganoderma* (southwest of the sampling point) this was not reflected in an increase in captured basidiospore concentrations. In contrast, the wind direction in the last six months of evaluation came from areas without positive detections of palms affected by *Ganoderma* (northeast of the sampling point); however, these months were characterized by the highest concentrations of basidiospores in the air. In any case, these results suggest that the increase in *Ganoderma* basidiospore concentrations in the air depends not only on the number and location of mature basidiocarps present during a specific period but also on the combination of favorable environmental conditions for their release and an appropriate wind speed for their dissemination.

## 5. Conclusions

Our study revealed that, while there are higher concentrations of *Ganoderma* basidiospores at heights below 4 m, there were no statistically significant differences with respect to captures made below 10 m. This suggests the possible origin of USR through basidiocarps at the base of affected palms in a local lot-level environment. However, it is emphasized that the success of any *Ganoderma* infection depends solely and exclusively on the occurrence and pairing of haploid basidiospores for the formation of infectious heterokaryons, which is more likely to occur in the soil than in the palm stem. Regarding the temporal capture pattern, an increase in basidiospore concentration was observed towards the end of the 12-month evaluation period, with a peak concentration of 3.7 × 10^2^ spores/m^3^. Additionally, a circadian rhythm of increased discharge was observed during nighttime compared to daytime, with a peak spore release at 2:00 a.m., possibly related to adaptive and evolutionary processes of the species. Nonetheless, the circadian rhythms of spore release seem to be well-defined by *Ganoderma* fungi. The variability in airborne basidiospore concentrations does not seem to be directly conditioned by environmental factors such as temperature, relative humidity or precipitation, suggesting that the spore discharge cycle may depend on multiple interactions between pathogen genetics and external environmental factors. Despite this, trends in correlation coefficients were observed that showed *Ganoderma* affinity for greater spore release during drier periods than wetter ones. Finally, a significant correlation was observed between wind speed and local airborne basidiospore concentration, especially at speeds below 1.5 m/s. However, it is discussed that values above these speeds could have contrary effects locally but significantly contribute to pathogen dissemination over long distances.

## Figures and Tables

**Table 1:** Spearman correlation coefficients between the concentration of basidiospores captured in the air and the meteorological factors recorded for each month from June 2022 to May 2023 ^a^.

Date		Solar Radiation	Temperature	Relative Humidity	Precipitation	Wind Speed at 2 m	Wind Speed at 10 m
Year	Month	(Mj/month)	(°C)	(%)	(mm)	(m/s)	(m/s)
2022	June	0.02	0.03	−0.01	−0.02	−0.18	−0.17
	July	0.13	0.13	−0.17	−0.25	0.04	0.13
	August	−0.16	−0.14	0.10	−0.01	0.07	−0.02
	September	−0.26	−0.41	0.22	−0.19	0.05	0.11
	October	0.32	0.01	−0.15	−0.03	0.16	0.14
	November	0.25	0.22	−0.18	−0.13	−0.06	−0.35
	December	0.02	0.28	−0.48	−0.25	−0.04	0.54 **
2023	January	0.15	0.17	−0.26	0.05	0.42 *	0.46 **
	February	0.21	0.23	−0.20	0.06	0.30	−0.29
	March	0.24	0.01	−0.07	0.15	0.24	0.22
	April	−0.15	−0.24	0.18	0.13	−0.04	−0.06
	May	0.01	0.24	−0.34	−0.18	−0.22	0.30

^a^ = * significance at *p* = 0.05, ** significance at *p* = 0.01.

## Data Availability

The raw data supporting the conclusions of this article will be made available by the authors on request.
